# *tert*-Butyl *N*-((1*S*)-2-hy­droxy-1-{*N*′-[(*E*)-2-hy­droxy-4-meth­oxy­benzyl­idene]hydrazinecarbon­yl}eth­yl)carbamate

**DOI:** 10.1107/S1600536811025293

**Published:** 2011-07-02

**Authors:** James L. Wardell, Marcus V. N. de Souza, Alessandra C. Pinheiro, Edward R. T. Tiekink, Solange M. S. V. Wardell

**Affiliations:** a Centro de Desenvolvimento Tecnológico em Saúde (CDTS), Fundação Oswaldo Cruz (FIOCRUZ), Casa Amarela, Campus de Manguinhos, Av. Brasil 4365, 21040-900 Rio de Janeiro, RJ, Brazil; b Fundação Oswaldo Cruz, Instituto de Tecnologia, em Fármacos – Farmanguinhos, R. Sizenando Nabuco, 100, Manguinhos, 21041-250 Rio de Janeiro, RJ, Brazil; c Department of Chemistry, University of Malaya, 50603 Kuala Lumpur, Malaysia; d CHEMSOL, 1 Harcourt Road, Aberdeen AB15 5NY, Scotland

## Abstract

The mol­ecule of the title compound, C_16_H_23_N_3_O_6_, is twisted about the chiral C atom with the dihedral angle formed between the amide residues being 76.9 (3)°. Overall, the mol­ecule is curved with the terminal organic groups lying to the same side. The conformation about the imine bond [1.291 (5) Å] is *E* and an intra­molecular O—H⋯N hydrogen bond generates an *S*(6) ring. In the crystal, O—H⋯O and N—H⋯O hydrogen bonds involving the hy­droxy, amine and carbonyl groups lead to the formation of supra­molecular layers, which stack along the *c*-axis direction.

## Related literature

For background to the use of l-serine derivatives in anti-tumour therapy, see: Jiao *et al.* (2009 ▶); Yakura *et al.* (2007 ▶). For background to *N*-acyl­hydrazone derivatives from l-serine for anti-tumour testing, see: Pinheiro *et al.* (2010 ▶, 2011*a* ▶,*b* ▶); de Souza *et al.* (2010 ▶, 2011 ▶); Howie *et al.* (2011 ▶); Tiekink *et al.* (2011 ▶).
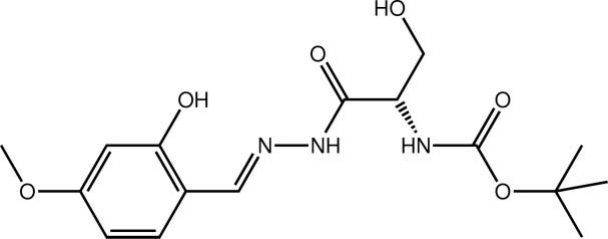


## Experimental

### 

#### Crystal data

C_16_H_23_N_3_O_6_
*M* *_r_* = 353.37
Triclinic, 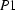

*a* = 5.3101 (14) Å
*b* = 5.7301 (13) Å
*c* = 14.651 (4) Å
α = 80.364 (16)°
β = 84.788 (11)°
γ = 74.845 (15)°
*V* = 423.70 (19) Å^3^
*Z* = 1
Mo *K*α radiation
μ = 0.11 mm^−1^
*T* = 120 K
0.62 × 0.18 × 0.03 mm

#### Data collection

Bruker–Nonius Roper CCD camera on κ-goniostat diffractometer
Absorption correction: multi-scan (*SADABS*; Sheldrick, 2007 ▶) *T*_min_ = 0.415, *T*_max_ = 0.746
7849 measured reflections
1936 independent reflections
1639 reflections with *I* > 2σ(*I*)
*R*_int_ = 0.092

#### Refinement

*R*[*F* ^2^ > 2σ(*F* ^2^)] = 0.062
*wR*(*F* ^2^) = 0.158
*S* = 1.06
1936 reflections
242 parameters
7 restraints
H atoms treated by a mixture of independent and constrained refinement
Δρ_max_ = 0.26 e Å^−3^
Δρ_min_ = −0.32 e Å^−3^

### 

Data collection: *COLLECT* (Hooft, 1998 ▶); cell refinement: *DENZO* (Otwinowski & Minor, 1997 ▶) and *COLLECT*; data reduction: *DENZO* and *COLLECT*; program(s) used to solve structure: *SHELXS97* (Sheldrick, 2008 ▶); program(s) used to refine structure: *SHELXL97* (Sheldrick, 2008 ▶); molecular graphics: *ORTEP-3* (Farrugia, 1997 ▶) and *DIAMOND* (Brandenburg, 2006 ▶); software used to prepare material for publication: *publCIF* (Westrip, 2010 ▶).

## Supplementary Material

Crystal structure: contains datablock(s) global, I. DOI: 10.1107/S1600536811025293/hb5936sup1.cif

Structure factors: contains datablock(s) I. DOI: 10.1107/S1600536811025293/hb5936Isup2.hkl

Additional supplementary materials:  crystallographic information; 3D view; checkCIF report

## Figures and Tables

**Table 1:** Hydrogen-bond geometry (Å, °)

*D*—H⋯*A*	*D*—H	H⋯*A*	*D*⋯*A*	*D*—H⋯*A*
O1—H1*o*⋯N1	0.84 (5)	1.99 (6)	2.666 (5)	137 (5)
O4—H4*o*⋯O3^i^	0.84 (3)	1.81 (3)	2.607 (5)	157 (6)
N2—H2*n*⋯O4^ii^	0.88 (4)	1.92 (3)	2.769 (5)	163 (4)
N3—H3*n*⋯O5^iii^	0.88 (4)	2.34 (4)	3.188 (5)	162 (4)

Symmetry codes: (i) 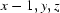; (ii) 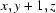; (iii) 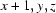.

**Fig. 1.**
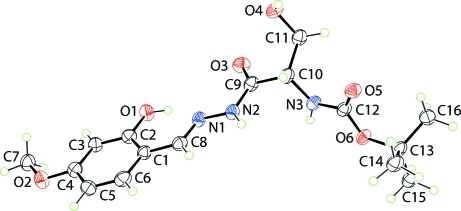
The molecular structure of (I) showing displacement ellipsoids at the 50% probability level.

**Fig. 2.**
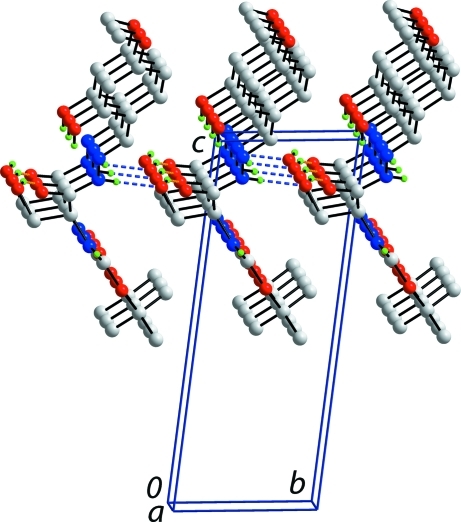
A view of the supramolecular array in the ab plane in (I) with the O—H···O and N—H···O hydrogen bonding shown as orange and blue dashed lines, respectively. Hydrogen atoms not participating in the hydrogen bonding scheme are omitted for reasons of clariy.

**Fig. 3.**
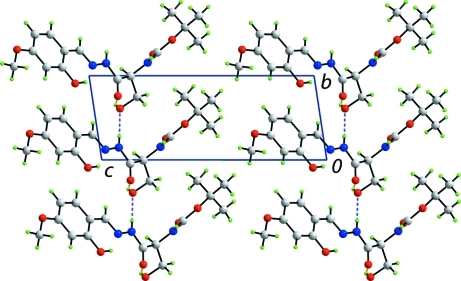
A view in projection down the a axis of the stacking of 2-D supramolecular arrays along the c axis in (I), and with the O—H···O and N—H···O hydrogen bonding shown as orange and blue dashed lines, respectively.

## supplementary crystallographic information

### Comment

The analysis of the title compound, (I), was conducted in the context of developing *N*-acylhydrazone derivatives from *L*-serine for use in anti-tumour testing (Pinheiro *et al.*, 2010; de Souza *et al.*, 2010; Pinheiro *et al.*, 2011*a*; Pinheiro *et al.*, 2011*b*; de Souza *et al.*, 2011; Howie *et al.*, 2011; Tiekink *et al.*, 2011) owing to the known anti-tumour activity of *L*-serine derivatives (Jiao *et al.*, 2009; Yakura *et al.*, 2007).

The absolute structure of (I) could not be determined experimentally but, the assignment of the *S*-configuration at the C10 atom is based on a starting reagent, *L*-serine. The structure of (I), Fig. 1, is isomorphous with the analogue not featuring the hydroxyl group in the ring (Pinheiro *et al.* 2011*b*). The molecule adopts a curved conformation with both the benzene ring and *tert*-butyl group lying to the same side of the molecule. Nevertheless, there is a twist in the molecule, at the chiral centre, as seen in the dihedral angle formed between the two amide residues, *i.e*. N2,C9,O3 and N3,C12,O5, of 76.9 (3) °. The presence of an intramolecular O—H···N hydrogen bond, Table 1, ensures that the hydroxybenzene group is co-planar with the adjacent hydrazine residue with the dihedral angle between the (O3,N1,N2,C8,C9) and (C1–C6) planes being 9.05 (14) °. The conformation about the N1═C8 imine bond [1.291 (5) Å] is *E*.

As with related structures in this series, hydrogen bonds dominate the crystal packing, Table 1. The secondary hydroxyl group forms a O—H···O hydrogen bond to the hydrazine-carbonyl-O2, and accepts a N—H···.O hydrogen bond from the hydrazine-amine, leading to chains along the *b* axis. The carbamate-amine forms a N—H···O hydrogen bond to the carbamate-carbonyl-O4, leading to chains along the *a* axis. The result is the formation of a two-dimensional array in the *ab* plane, Fig. 2. The layers stack along the *c* axis, Fig. 3.

### Experimental

To a stirred solution of (*S*)-t-BuOCONHCH(CH_2_OH)CONHNH_2_ (Howie *et al.*, 2011) (1.0 mmol) in ethanol (10 ml) at room temperature was added 2-hydroxy-4-methoxybenzaldehyde (1.05 mmol). The reaction mixture was refluxed for 4 h, rotary evaporated and the residue purified by washing with cold ethanol (3 *x* 10 ml), affording the title compound, *M*.pt. 455 K, yield 84%. The sample for the structure determination was recrystallized from EtOH to afford colourless laths of (I).

^1^H NMR (500 MHz, DMSO-d6) δ (p.p.m.): 11.60 (1*H*, s, NHN), 11.46 (1*H*, s, C1—OH), 8.35 (1*H*, s, N=CH), 7.39 (1*H*, d, J = 8.4, H6), 6.84 (1*H*, d, J = 7.4, NHCH), 6.55–6.40 (2*H*, m, H3 and H5), 4.98 (1*H*, m, OH), 4.02 (1*H*, m, CH), 3.76 (3*H*, s, CH_3_O), 3.70–3.50 (2*H*, m, CH_2_OH), 1.39 (9*H*, s, (CH_3_)_3_C). ^13^C NMR(125 MHz, DMSO-d6) δ (p.p.m.): 171.5, 162.5, 158.3, 155.7, 141.8, 128.3, 112.1, 106.9, 101.4, 78.5, 61.6, 56.3, 55.6, 28.6. IR (cm^-1^, KBr): 3375 (O—H), 1677 (COCH and COO). MS/ESI: [M—H]: 352.4.

### Refinement

The C-bound H atoms were geometrically placed (C–H = 0.95–1.00 Å) and refined as riding with *U*_iso_(H) = 1.2–1.5*U*_eq_(C). The O– and N-bound H atoms were located from a difference map and refined with the distance restraints O–H = 0.84 ± 0.01 and N–H = 0.88±0.01 Å, and with *U*_iso_(H) = *zU*_eq_(carrier atom); *z* = 1.5 for O and *z* = 1.2 for N. In the absence of significant anomalous scattering effects, 1703 Friedel pairs were averaged in the final refinement. However, the absolute configuration was assigned on the basis of the chirality of the *L*-serine starting material.

### Crystal data

**Table:**

C_16_H_23_N_3_O_6_	*Z* = 1
*M**_r_* = 353.37	*F*(000) = 188
Triclinic, *P*1	*D*_x_ = 1.385 Mg m^−^^3^
Hall symbol: P 1	Mo *K*α radiation, λ = 0.71073 Å
*a* = 5.3101 (14) Å	Cell parameters from 41272 reflections
*b* = 5.7301 (13) Å	θ = 2.9–27.5°
*c* = 14.651 (4) Å	µ = 0.11 mm^−^^1^
α = 80.364 (16)°	*T* = 120 K
β = 84.788 (11)°	Lath, colourless
γ = 74.845 (15)°	0.62 × 0.18 × 0.03 mm
*V* = 423.70 (19) Å^3^	

### Data collection

**Table:**

Bruker–Nonius Roper CCD camera on κ-goniostat diffractometer	1936 independent reflections
Radiation source: Bruker–Nonius FR591 rotating anode	1639 reflections with *I* > 2σ(*I*)
graphite	*R*_int_ = 0.092
Detector resolution: 9.091 pixels mm^-1^	θ_max_ = 27.5°, θ_min_ = 3.7°
φ & ω scans	*h* = −6→6
Absorption correction: multi-scan (*SADABS*; Sheldrick, 2007)	*k* = −7→7
*T*_min_ = 0.415, *T*_max_ = 0.746	*l* = −18→18
7849 measured reflections	

### Refinement

**Table:**

Refinement on *F*^2^	Secondary atom site location: difference Fourier map
Least-squares matrix: full	Hydrogen site location: inferred from neighbouring sites
*R*[*F*^2^ > 2σ(*F*^2^)] = 0.062	H atoms treated by a mixture of independent and constrained refinement
*wR*(*F*^2^) = 0.158	*w* = 1/[σ^2^(*F*_o_^2^) + (0.0932*P*)^2^ + 0.0716*P*] where *P* = (*F*_o_^2^ + 2*F*_c_^2^)/3
*S* = 1.06	(Δ/σ)_max_ < 0.001
1936 reflections	Δρ_max_ = 0.26 e Å^−^^3^
242 parameters	Δρ_min_ = −0.32 e Å^−^^3^
7 restraints	Absolute structure: nd
Primary atom site location: structure-invariant direct methods	

### Special details

**Table:**

Geometry. All s.u.'s (except the s.u. in the dihedral angle between two l.s. planes) are estimated using the full covariance matrix. The cell s.u.'s are taken into account individually in the estimation of s.u.'s in distances, angles and torsion angles; correlations between s.u.'s in cell parameters are only used when they are defined by crystal symmetry. An approximate (isotropic) treatment of cell s.u.'s is used for estimating s.u.'s involving l.s. planes.
Refinement. Refinement of *F*^2^ against ALL reflections. The weighted *R*-factor *wR* and goodness of fit *S* are based on *F*^2^, conventional *R*-factors *R* are based on *F*, with *F* set to zero for negative *F*^2^. The threshold expression of *F*^2^ > 2σ(*F*^2^) is used only for calculating *R*-factors(gt) *etc*. and is not relevant to the choice of reflections for refinement. *R*-factors based on *F*^2^ are statistically about twice as large as those based on *F*, and *R*- factors based on ALL data will be even larger.

### Fractional atomic coordinates and isotropic or equivalent isotropic displacement parameters (Å^2^)

**Table:**

	*x*	*y*	*z*	*U*_iso_*/*U*_eq_	
O1	1.3235 (6)	−0.0932 (5)	1.0670 (2)	0.0337 (7)	
H1o	1.233 (10)	−0.078 (11)	1.021 (3)	0.051*	
O2	1.5080 (6)	0.3094 (6)	1.3026 (2)	0.0365 (7)	
O3	0.8525 (6)	−0.2427 (5)	0.8860 (2)	0.0359 (7)	
O4	0.3605 (6)	−0.3907 (5)	0.8847 (2)	0.0330 (7)	
H4o	0.201 (3)	−0.351 (11)	0.901 (4)	0.049*	
O5	0.0570 (6)	0.2513 (5)	0.7006 (2)	0.0318 (7)	
O6	0.3497 (6)	0.4260 (5)	0.6066 (2)	0.0307 (7)	
N1	0.8884 (7)	0.1219 (6)	0.9773 (2)	0.0296 (8)	
N2	0.6946 (7)	0.1467 (6)	0.9162 (2)	0.0289 (7)	
H2n	0.564 (7)	0.277 (6)	0.907 (3)	0.035*	
N3	0.4886 (7)	0.1547 (6)	0.7304 (2)	0.0290 (8)	
H3n	0.630 (6)	0.207 (9)	0.712 (3)	0.035*	
C1	1.0379 (8)	0.2948 (7)	1.0923 (3)	0.0277 (8)	
C2	1.2576 (8)	0.1000 (7)	1.1135 (3)	0.0296 (9)	
C3	1.4175 (8)	0.0995 (7)	1.1835 (3)	0.0285 (8)	
H3	1.5648	−0.0341	1.1972	0.034*	
C4	1.3625 (8)	0.2931 (8)	1.2332 (3)	0.0311 (9)	
C5	1.1485 (10)	0.4908 (8)	1.2127 (3)	0.0360 (10)	
H5	1.1141	0.6261	1.2456	0.043*	
C6	0.9868 (9)	0.4896 (8)	1.1443 (3)	0.0351 (10)	
H6	0.8381	0.6226	1.1320	0.042*	
C7	1.7297 (9)	0.1110 (9)	1.3266 (3)	0.0380 (10)	
H7A	1.6729	−0.0403	1.3454	0.057*	
H7B	1.8147	0.1440	1.3779	0.057*	
H7C	1.8534	0.0933	1.2728	0.057*	
C8	0.8536 (9)	0.2999 (8)	1.0244 (3)	0.0313 (9)	
H8	0.7059	0.4353	1.0146	0.038*	
C9	0.6886 (8)	−0.0437 (7)	0.8759 (3)	0.0298 (9)	
C10	0.4544 (8)	−0.0039 (7)	0.8164 (3)	0.0283 (9)	
H10	0.2931	0.0778	0.8509	0.034*	
C11	0.4262 (9)	−0.2510 (7)	0.7997 (3)	0.0322 (9)	
H11A	0.2880	−0.2258	0.7554	0.039*	
H11B	0.5923	−0.3421	0.7720	0.039*	
C12	0.2788 (8)	0.2769 (7)	0.6807 (3)	0.0279 (8)	
C13	0.1569 (8)	0.5755 (7)	0.5405 (3)	0.0296 (9)	
C14	−0.0560 (9)	0.7519 (8)	0.5888 (3)	0.0353 (10)	
H14A	−0.1694	0.6616	0.6280	0.053*	
H14B	−0.1596	0.8736	0.5424	0.053*	
H14C	0.0236	0.8347	0.6274	0.053*	
C15	0.3213 (9)	0.7096 (8)	0.4718 (3)	0.0347 (10)	
H15A	0.4001	0.8072	0.5042	0.052*	
H15B	0.2106	0.8176	0.4239	0.052*	
H15C	0.4595	0.5908	0.4428	0.052*	
C16	0.0458 (9)	0.4084 (8)	0.4931 (3)	0.0337 (9)	
H16A	0.1896	0.2843	0.4694	0.051*	
H16B	−0.0583	0.5058	0.4416	0.051*	
H16C	−0.0651	0.3282	0.5380	0.051*	

### Atomic displacement parameters (Å^2^)

**Table:**

	*U*^11^	*U*^22^	*U*^33^	*U*^12^	*U*^13^	*U*^23^
O1	0.0317 (16)	0.0303 (15)	0.0357 (16)	0.0037 (12)	−0.0057 (12)	−0.0114 (12)
O2	0.0309 (17)	0.0413 (18)	0.0365 (17)	−0.0023 (13)	−0.0052 (13)	−0.0125 (13)
O3	0.0266 (16)	0.0257 (15)	0.0500 (19)	0.0022 (12)	−0.0051 (13)	−0.0032 (13)
O4	0.0271 (15)	0.0236 (14)	0.0406 (17)	0.0028 (12)	−0.0029 (13)	0.0025 (12)
O5	0.0281 (16)	0.0255 (15)	0.0386 (17)	−0.0033 (11)	−0.0039 (12)	−0.0002 (12)
O6	0.0267 (15)	0.0273 (14)	0.0327 (15)	0.0000 (11)	−0.0064 (12)	0.0023 (11)
N1	0.0277 (18)	0.0285 (18)	0.0288 (17)	−0.0005 (14)	−0.0054 (13)	−0.0018 (14)
N2	0.0253 (18)	0.0279 (18)	0.0296 (17)	0.0007 (13)	−0.0051 (14)	−0.0027 (14)
N3	0.0272 (18)	0.0242 (17)	0.0315 (18)	−0.0005 (14)	−0.0037 (14)	−0.0007 (14)
C1	0.028 (2)	0.025 (2)	0.028 (2)	−0.0023 (16)	−0.0034 (15)	−0.0022 (15)
C2	0.030 (2)	0.025 (2)	0.031 (2)	−0.0014 (17)	0.0016 (17)	−0.0060 (16)
C3	0.026 (2)	0.0257 (19)	0.031 (2)	0.0015 (15)	−0.0026 (16)	−0.0059 (15)
C4	0.031 (2)	0.032 (2)	0.028 (2)	−0.0038 (17)	0.0016 (17)	−0.0050 (17)
C5	0.045 (3)	0.029 (2)	0.033 (2)	−0.0061 (19)	−0.0011 (19)	−0.0093 (17)
C6	0.035 (2)	0.025 (2)	0.038 (2)	0.0014 (17)	−0.0014 (18)	−0.0016 (17)
C7	0.039 (3)	0.036 (2)	0.035 (2)	−0.002 (2)	−0.0066 (19)	−0.0059 (19)
C8	0.031 (2)	0.027 (2)	0.033 (2)	−0.0033 (17)	−0.0044 (17)	−0.0004 (16)
C9	0.028 (2)	0.0241 (19)	0.031 (2)	0.0029 (16)	−0.0033 (16)	−0.0017 (15)
C10	0.027 (2)	0.0256 (19)	0.028 (2)	0.0012 (16)	−0.0030 (16)	−0.0021 (15)
C11	0.034 (2)	0.027 (2)	0.032 (2)	0.0005 (17)	−0.0032 (18)	−0.0045 (17)
C12	0.030 (2)	0.0211 (18)	0.029 (2)	0.0001 (15)	−0.0033 (16)	−0.0017 (15)
C13	0.031 (2)	0.0217 (19)	0.031 (2)	0.0020 (16)	−0.0042 (16)	−0.0005 (15)
C14	0.039 (2)	0.026 (2)	0.035 (2)	0.0025 (18)	−0.0022 (18)	−0.0044 (17)
C15	0.036 (2)	0.028 (2)	0.035 (2)	0.0008 (18)	−0.0037 (18)	−0.0020 (17)
C16	0.034 (2)	0.028 (2)	0.037 (2)	−0.0026 (17)	−0.0051 (18)	−0.0062 (17)

### Geometric parameters (Å, °)

**Table:**

O1—C2	1.348 (5)	C5—C6	1.379 (7)
O1—H1o	0.842 (11)	C5—H5	0.9500
O2—C4	1.362 (5)	C6—H6	0.9500
O2—C7	1.429 (5)	C7—H7A	0.9800
O3—C9	1.236 (5)	C7—H7B	0.9800
O4—C11	1.432 (5)	C7—H7C	0.9800
O4—H4o	0.841 (11)	C8—H8	0.9500
O5—C12	1.228 (5)	C9—C10	1.530 (6)
O6—C12	1.353 (5)	C10—C11	1.523 (6)
O6—C13	1.472 (5)	C10—H10	1.0000
N1—C8	1.291 (5)	C11—H11A	0.9900
N1—N2	1.388 (5)	C11—H11B	0.9900
N2—C9	1.333 (5)	C13—C14	1.516 (6)
N2—H2n	0.880 (10)	C13—C15	1.516 (6)
N3—C12	1.353 (5)	C13—C16	1.529 (6)
N3—C10	1.452 (5)	C14—H14A	0.9800
N3—H3n	0.882 (11)	C14—H14B	0.9800
C1—C2	1.404 (5)	C14—H14C	0.9800
C1—C6	1.410 (6)	C15—H15A	0.9800
C1—C8	1.449 (6)	C15—H15B	0.9800
C2—C3	1.389 (6)	C15—H15C	0.9800
C3—C4	1.381 (6)	C16—H16A	0.9800
C3—H3	0.9500	C16—H16B	0.9800
C4—C5	1.393 (6)	C16—H16C	0.9800
			
C2—O1—H1o	114 (4)	N2—C9—C10	115.1 (3)
C4—O2—C7	117.7 (3)	N3—C10—C11	112.1 (3)
C11—O4—H4o	114 (4)	N3—C10—C9	109.5 (3)
C12—O6—C13	121.0 (3)	C11—C10—C9	109.2 (3)
C8—N1—N2	114.7 (3)	N3—C10—H10	108.6
C9—N2—N1	119.3 (3)	C11—C10—H10	108.6
C9—N2—H2n	117 (3)	C9—C10—H10	108.6
N1—N2—H2n	124 (3)	O4—C11—C10	110.7 (3)
C12—N3—C10	119.8 (3)	O4—C11—H11A	109.5
C12—N3—H3n	113 (3)	C10—C11—H11A	109.5
C10—N3—H3n	126 (3)	O4—C11—H11B	109.5
C2—C1—C6	117.6 (4)	C10—C11—H11B	109.5
C2—C1—C8	123.9 (4)	H11A—C11—H11B	108.1
C6—C1—C8	118.4 (4)	O5—C12—N3	123.8 (4)
O1—C2—C3	117.1 (3)	O5—C12—O6	125.6 (4)
O1—C2—C1	121.9 (4)	N3—C12—O6	110.5 (4)
C3—C2—C1	121.0 (4)	O6—C13—C14	110.6 (3)
C4—C3—C2	120.1 (4)	O6—C13—C15	102.4 (3)
C4—C3—H3	120.0	C14—C13—C15	111.4 (3)
C2—C3—H3	120.0	O6—C13—C16	109.5 (3)
O2—C4—C3	124.3 (4)	C14—C13—C16	111.9 (4)
O2—C4—C5	115.4 (4)	C15—C13—C16	110.7 (4)
C3—C4—C5	120.2 (4)	C13—C14—H14A	109.5
C6—C5—C4	119.8 (4)	C13—C14—H14B	109.5
C6—C5—H5	120.1	H14A—C14—H14B	109.5
C4—C5—H5	120.1	C13—C14—H14C	109.5
C5—C6—C1	121.3 (4)	H14A—C14—H14C	109.5
C5—C6—H6	119.3	H14B—C14—H14C	109.5
C1—C6—H6	119.3	C13—C15—H15A	109.5
O2—C7—H7A	109.5	C13—C15—H15B	109.5
O2—C7—H7B	109.5	H15A—C15—H15B	109.5
H7A—C7—H7B	109.5	C13—C15—H15C	109.5
O2—C7—H7C	109.5	H15A—C15—H15C	109.5
H7A—C7—H7C	109.5	H15B—C15—H15C	109.5
H7B—C7—H7C	109.5	C13—C16—H16A	109.5
N1—C8—C1	120.5 (4)	C13—C16—H16B	109.5
N1—C8—H8	119.7	H16A—C16—H16B	109.5
C1—C8—H8	119.7	C13—C16—H16C	109.5
O3—C9—N2	124.3 (4)	H16A—C16—H16C	109.5
O3—C9—C10	120.6 (4)	H16B—C16—H16C	109.5
			
C8—N1—N2—C9	−168.9 (4)	C6—C1—C8—N1	−177.9 (4)
C6—C1—C2—O1	−179.4 (4)	N1—N2—C9—O3	−3.1 (6)
C8—C1—C2—O1	4.4 (6)	N1—N2—C9—C10	175.1 (3)
C6—C1—C2—C3	0.3 (6)	C12—N3—C10—C11	79.4 (4)
C8—C1—C2—C3	−175.8 (4)	C12—N3—C10—C9	−159.2 (3)
O1—C2—C3—C4	179.4 (4)	O3—C9—C10—N3	−107.6 (4)
C1—C2—C3—C4	−0.4 (6)	N2—C9—C10—N3	74.1 (4)
C7—O2—C4—C3	−1.1 (6)	O3—C9—C10—C11	15.5 (5)
C7—O2—C4—C5	179.9 (4)	N2—C9—C10—C11	−162.8 (4)
C2—C3—C4—O2	−179.7 (4)	N3—C10—C11—O4	−172.8 (3)
C2—C3—C4—C5	−0.8 (6)	C9—C10—C11—O4	65.6 (4)
O2—C4—C5—C6	−179.0 (4)	C10—N3—C12—O5	−4.8 (6)
C3—C4—C5—C6	1.9 (7)	C10—N3—C12—O6	176.0 (3)
C4—C5—C6—C1	−2.0 (7)	C13—O6—C12—O5	−0.4 (6)
C2—C1—C6—C5	0.9 (7)	C13—O6—C12—N3	178.8 (3)
C8—C1—C6—C5	177.2 (4)	C12—O6—C13—C14	60.8 (5)
N2—N1—C8—C1	178.5 (4)	C12—O6—C13—C15	179.6 (3)
C2—C1—C8—N1	−1.8 (7)	C12—O6—C13—C16	−62.9 (4)

### Hydrogen-bond geometry (Å, °)

**Table:**

*D*—H···*A*	*D*—H	H···*A*	*D*···*A*	*D*—H···*A*
O1—H1o···N1	0.84 (5)	1.99 (6)	2.666 (5)	137 (5)
O4—H4o···O3^i^	0.84 (3)	1.81 (3)	2.607 (5)	157 (6)
N2—H2n···O4^ii^	0.88 (4)	1.92 (3)	2.769 (5)	163 (4)
N3—H3n···O5^iii^	0.88 (4)	2.34 (4)	3.188 (5)	162 (4)

Symmetry codes: (i) *x*−1, *y*, *z*; (ii) *x*, *y*+1, *z*; (iii) *x*+1, *y*, *z*.
